# Annexin A3 as a Prognostic Biomarker for Breast Cancer: A Retrospective Study

**DOI:** 10.1155/2017/2603685

**Published:** 2017-04-13

**Authors:** Tao Zhou, Yong Li, Li Yang, Tiantian Tang, Lina Zhang, Jiajie Shi

**Affiliations:** ^1^Department of Breast Cancer Center, The Fourth Hospital, Hebei Medical University, Shijiazhuang, Hebei 050011, China; ^2^Department of General Surgery, The Fourth Hospital, Hebei Medical University, Shijiazhuang, Hebei 050011, China; ^3^Department of CT, The Fourth Hospital, Hebei Medical University, Shijiazhuang, Hebei 050011, China

## Abstract

To validate the correlation between ANXA3 expression and prognosis in breast cancer, a retrospective study encompassing 309 breast cancer patients was performed. The expression of ANXA3 was determined by the immunohistochemical examination of tissue sections by the Max Vision™ method. The ANXA3 levels in the patient samples were validated for the prognosis based on age, menopause status, tumor size, tumor node, metastasis stage, the number of lymphatic metastases, oncology grade, and molecular subtyping. An elevated expression of ANXA3 was detected in breast cancer samples, compared to adjacent tissue samples, and significant correlation depending on the number of lymphatic metastases (*P* = 0.001) and histological grade (*P* = 0.004) was observed. The number of lymphatic metastases and ANXA3 expression were identified as independent risk factors affecting the disease-free survival and overall survival. Significantly (*P* < 0.002) higher level of ANXA3 was detected in triple-negative breast cancer compared to other subtypes. There was no significant (*P* > 0.05) change in the expression of ANXA3 with respect to age, menopausal status, tumor size, and clinical stage. The findings implicate the expression of ANXA3 with the natural progression of breast cancer and associate it with increased lymphatic metastasis. The study validates the use of ANXA3 as a potential prognosis biomarker for breast cancer.

## 1. Introduction

Annexin is highly conserved and ubiquitously distributed in various body cell types and accounts for about 0.5–2% of the total cell protein [1, 2]. It is involved in membrane transport and a series of calmodulin-dependent activities on the surface of the cell membrane, including vesicular transport, membrane fusion in exocytosis, signal transduction, and formation of calcium channels, and also regulates inflammatory response, cell differentiation, and interaction between cytoskeletal proteins [3, 4]. Dysregulation of annexin is associated with the development and progression of different types of cancer [5, 6].

The human genome encodes for 12 different annexins varying in expression and distribution within the tissues. Some of these are ubiquitously expressed (A1, A2, A5, A6, and A7), while some are selective (A3, A8, A9, A10, and A13). The differential expression of annexin A3 (ANXA3) plays a major role in tumorigenesis, drug resistance, and metastasis [7]. The ANXA3 gene is located on human chromosome 4q13-q22 and encodes a protein of 323 amino acid residues [7]. Levels of ANXA3 expression have shown significant differences in colorectal cancer [8, 9], pancreatic cancer [9–11], lung cancer [12], and hepatocellular carcinoma [13]. Breast cancer is the second most commonly diagnosed malignancy and a leading cause of cancer-related death in women worldwide [14]. Novel candidates as prognostic biomarkers need evaluation for efficacy through multiple studies before their routine use in diagnostic and treatment procedures for breast cancer. Therefore, in this study, immunohistochemical investigation for differential expression of ANXA3 in breast cancer tissue of different subtypes was determined to validate its correlation with the clinicopathological features and prognosis of breast cancer.

## 2. Patients and Methods

### 2.1. Patients

The approval for the retrospective study was obtained from the Ethics Committee of the Fourth Hospital of Hebei Medical University. The study included 309 breast cancer patients admitted to the hospital between January and December 2009.

*Inclusion Criteria.* (1) Patients were pathologically confirmed with breast cancer, based on the 6th edition of the AJCC Cancer Staging Manual [15]; (2) age range was 18–80 years; (3) the patients must have received standard surgery and/or radiochemotherapy and completed the treatment protocol according to NCCN (National Comprehensive Cancer Network) Clinical Practice Guidelines in Breast Cancer (2008).

*Exclusion Criteria.* (1) Patients with recurrent, metastatic, or topical advanced breast cancer and (2) patients with incomplete follow-up data were excluded.

### 2.2. Immunohistochemistry

Immunohistochemistry was performed using the Max Vision method. The primary antibodies for ER (SP1) and PR (SP2) were purchased from the Fuzhou Maixin Biotech. Co., Ltd. (Fuzhou, China). The primary antibodies for HER-2 (4B5) and Ki-67 (30-9) were procured from Roche Pharmaceuticals Ltd. (Shanghai, China). Annexin A3 primary antibodies (ab127924) were obtained from Abcam. PBS was used as the negative control in place of the primary antibody. The positive control was performed following the manufacturer's guidelines. The specimens were paraffin-embedded and sliced into sections (4 *μ*M), washed thrice (2 min) with distilled water, and treated with 3% hydrogen peroxide (10 min) to block the endogenous peroxidase. After incubation, the sections were washed thrice (2 min) with distilled water and placed in a pressure cooker for heat-induced epitope retrieval. After the heat treatment, the sections were washed thrice (5 min) with phosphate-buffered saline (PBS, pH 7.4) and incubated with 4% goat serum at room temperature for 30 min to reduce nonspecific staining. Subsequently, specimens were incubated with the primary antibody for 1 h, washed thrice (5 min) with PBS, incubated with Max Vision HPR antibody for 15 min, washed thrice (5 min) with PBS, and developed with freshly prepared DAB. The stained sections were observed under a microscope; the positive signal was clay bank or brown.

As a routine procedure, the cells were developed for about 5 min before the reaction was terminated and were fully washed using running water, counterstained with hematoxylin, differentiated using 0.1% hydrochloric-alcohol solution, and washed using running water, followed by dehydration with gradient ethanol, vitrification with dimethylbenzene, and mounting with neutral balsam, and the images were recorded.

### 2.3. Pathological Evaluation

Two experienced chief physicians reviewed the results of pathological examinations.

*Criteria for Determining ER/PR [16, 17].* In accordance with the immunohistochemistry results described earlier, cases with clay bank cancer nuclei accounting for <10% and ≥10% of all the nuclei were defined as negative and positive ER/PR expressions, respectively. 

**Figure 1:**
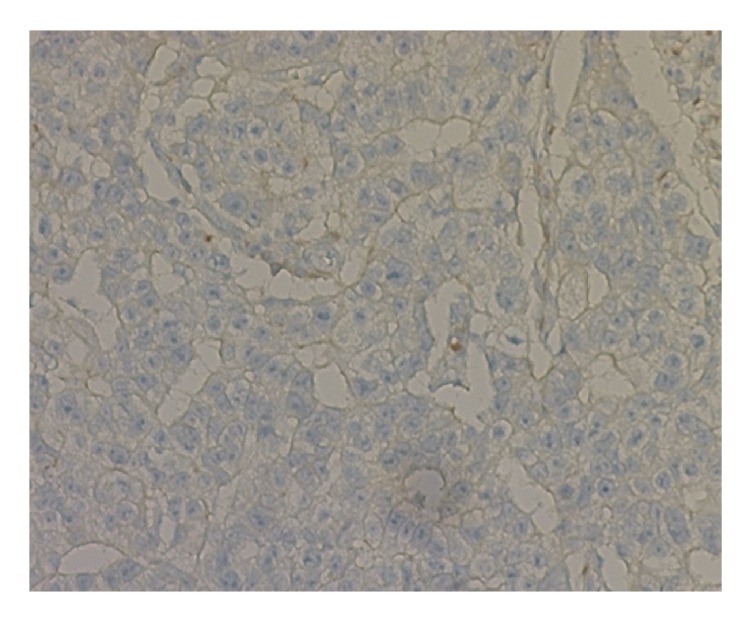
Representative stained histochemical sections negative for ANXA3 (×20).

**Figure 2:**
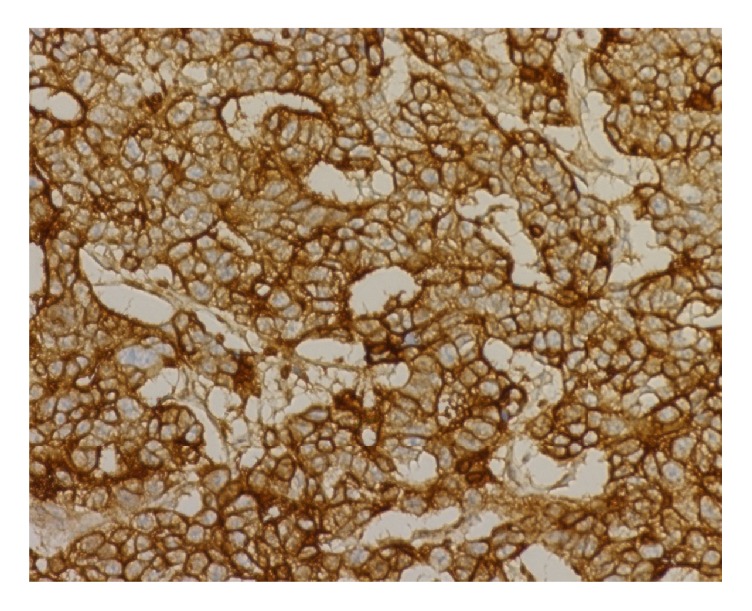
Representative stained histochemical sections strongly positive for ANXA3 (×40). Immunohistochemistry results were evaluated based on the percentage of positively stained cells and the staining intensity.

*Criteria for Determining ANXA3-Positive Expression.* ANXA3 is dominated by cytoplasmic staining. Immunohistochemistry results were evaluated based on the percentage of positively stained cells and the staining intensity [18]: (1) number of positive cells: 5 high-power fields were randomly selected from each section for counting the number of positively stained cells, where 1–24%, 25–49%, 50–74%, and >75% were referred to as 1, 2, 3, and 4 points, respectively; (2) staining intensity: 0 referred to the absence of stained cells, 1 referred to faint yellow granules that were significantly higher than the background, 2 referred to light brownish yellow granules, and 3 referred to a large number of dark brownish yellow granules. Summation of staining intensity score and percentage of positive cells score < 2 was referred to as negative (−), while a summation > 2 was positive, of which 2-3, 4-5, and 6-7 points referred to weakly positive (+), moderately positive (++), and strongly positive (+++), respectively. Representative immunohistochemical staining image is provided in Figures 1 and 2. 

*Criteria for Determining HER-2 [19].* The criteria included the following: (1) the percentage of cancer cells with fully stained membranes as well as the staining intensity must be focused on, (2) cells with stained cytoplasm were excluded, (3) only infiltrating carcinoma results were evaluated, while those of intraductal carcinomas were excluded, (4) normal mammary gland epithelial cells should not be stained. The HercepTest™ criteria recommended by US FDA were adopted [20–22]: for each section, results were classified into (−)–(+++), where (−) referred to no stained cells or cancer cells with stained membrane accounting for <10% of all the cancer cells, (+) referred to cancer cells with lightly and incompletely stained membrane accounting for >10% of all the cancer cells, and (++) referred to cancer cells with lightly to moderately and completely stained membrane accounting for >10% of all the cancer cells, whereas (+++) referred to cancer cells with strongly and completely stained membrane accounting for 10% of all the cells. Cases with (−) were considered to have no expression, those with (+) were considered to have low expression, and those with (+++) were considered to have high expression, while cases with (++) were selected to undergo a FISH test with kit (CPD0009) purchased from Beijing GP Medical Technologies, Ltd. (Beijing, China). A ratio of the total number of red signals over the total number of green signals ≥ 2 referred to HER-2 gene amplification; otherwise it was considered as absence of amplification. Cases with HER-2 amplification were considered to have high expression, while those lacking HER-2 amplification were considered to have low expression. 

*Criteria for Determining Ki-67 [23].* Cells with brown stained nuclei were found to be positive. The results were assessed by calculating the percentages of positive cells in 5 high-power fields, where <10% was denoted as (−), 10–20% was denoted as (+), and >20% was denoted as (++).

### 2.4. Prognosis

From the day of pathological diagnosis with breast cancer and the start date of formal treatment, patients were followed up every 6 months until June 2015. Patients were considered to have local recurrence if the cancer cells were pathologically confirmed in topical or regional lymph nodes. Moreover, the patients were deemed to have distant metastasis if radiology or pathology confirmed the metastasis in lung, liver, or bone, as well as contralateral breast, axillary or supraclavicular lymph nodes, and other sites. Disease-free survival (DFS) referred to the first day of treatment to local recurrence or distant metastasis. The overall survival (OS) implied the period from the first day of therapy to breast cancer-induced death or last follow-up. The patient data was retrospectively examined for age, menopause status, tumor size, TNM (tumor node and metastasis) stage, the number of lymphatic metastases, oncology grade, and molecular subtyping.

### 2.5. Statistical Analysis

The statistical analysis was performed using the SPSS 13.0 (IBM, USA) software. Comparisons between the groups were conducted using *χ*^2^ test. DFS and OS were calculated using Kaplan-Meier method, while comparisons of curves between the two groups were performed using the log-rank test. The level of significance was determined as *P* < 0.05.

## 3. Results

### 3.1. Patient Characteristics

The 309 patients with primary breast cancer enrolled for the study were of the age range of 22–76 years with a median age of 47 years. Based on the TNM (tumor node and metastasis) staging described in the 6th edition of the AJCC Cancer Staging Manual (2003) [15], there were 58 stage I, 219 stage II, and 32 stage III cases of breast cancer. In terms of the pathological type, there were 202 infiltrating ductal carcinomas, 35 infiltrating lobular carcinomas, 29 medullary carcinomas, and 43 other types of carcinomas (13 mucinous adenocarcinoma, 10 poorly differentiated adenocarcinomas, 7 lipid-rich carcinomas, 5 squamous carcinomas, 5 canalicular adenomas, and 3 adenoacanthoma-cell carcinoma cases). The cohort of 309 breast cancer patients consisted of 72 cases with axillary lymphatic metastasis > 3 pieces, accounting for 22%. Based on the immunohistochemistry data, ER+ or PR+ was detected in 234 cases, accounting for 75.73%.

### 3.2. Correlation between ANXA3 Protein Expression and Clinicopathological Indexes

Among the 309 patients, 193 (62.4%) were positive for the expression of ANXA3. With the increase in the histological grade, there was significant (*χ*^2^ = 16.686; *P* < 0.001) increase in the cases positive for the expression of ANXA3. In 72 patients with positive axillary nodes > 3, the expression of ANXA3 (73.6%) was significantly (*χ*^2^ = 11.123; *P* = 0.004) higher as compared to patients with negative axillary nodes (51.94%). Further subgroup analyses revealed that the positive expression rate of ANXA3 in patients with triple-negative breast cancer (TNBC) (79.66%) was significantly higher than that in patients with other types of breast cancer (*χ*^2^ = 9.226; *P* = 0.026). However, the differences in the expression of ANXA3 between other subgroups were not statistically significant (*χ*^2^ = 0.022; *P* = 0.989). In the present study, age, menopausal status, tumor size, and clinical stage did not show significant differences between the two groups (*χ*^2^ = 0.365 and *P* = 0.546; *χ*^2^ = 1.560 and *P* = 0.240; *χ*^2^ = 1.532 and *P* = 0.465; *χ*^2^ = 0.607 and *P* = 0.738) (Table 1).

### 3.3. Follow-Up

**Figure 3:**
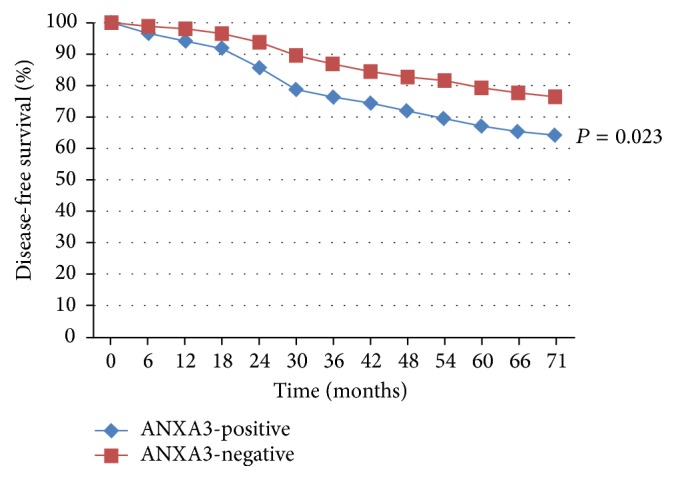
Kaplan-Meier survival curves for DFS. Expression of ANXA3 as estimated from immunohistochemical analysis correlated to retrospective data on DFS. ANXA3 expression is a significant prognostic factor for DFS. *χ*^2^ = 5.265; *P* = 0.023.

**Figure 4:**
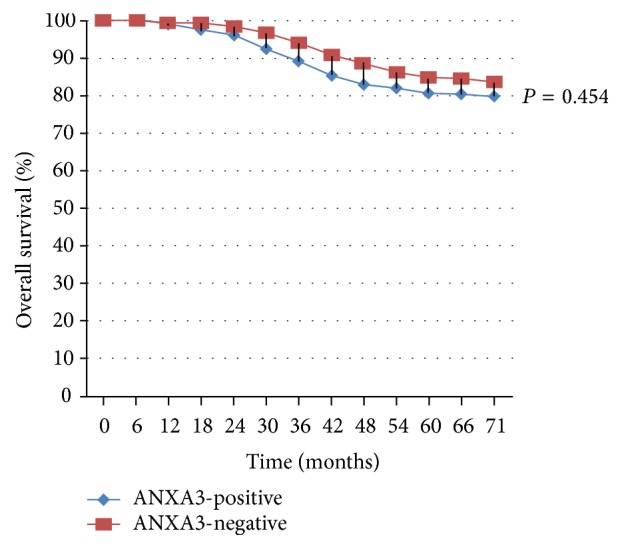
Kaplan-Meier survival curves for OS. Expression of ANXA3 as estimated from immunohistochemical analysis correlated to retrospective data on OS. *χ*^2^ = 0.696; *P* = 0.454.

Postoperative patients were followed up for 66–78 months, with a median of 71 months. In 96 patients, local recurrence or distant metastasis was observed, and their 71-month DFS was 68.93% (213/309), with a significant (*χ*^2^ = 5.265; *P* = 0.023) difference between the ANXA3-positive (64.25%, 124/193) and ANXA3-negative (76.72%, 89/116) expression groups (Figure 3). There were 58 cases of death, and their 71-month OS was 81.23% (251/309), with a significant (*χ*^2^ = 0.696; *P* = 0.454) difference between the ANXA3-positive (79.79%, 154/193) and ANXA3-negative (83.62%, 97/116) expression groups (Figure 4).

### 3.4. Multivariate Cox Analysis for Factors Affecting DFS and OS of Patients with Breast Cancer

Multivariate Cox analysis was performed with the variables including tumor size, TNM stage, the number of lymphatic metastases, oncology grade, molecular subtyping, ANXA3 expression, and other indexes (all with *P* values < 0.10 in univariate analyses). The multivariate Cox analysis correlated the levels of ANXA3 expression to retrospectively examined DFS (Figure 3) and OS (Figure 4). The analysis revealed that the tumor size, TNM stage, oncology grade, and molecular subtyping were not significantly (*P* > 0.05) correlated with DFS and OS. However, the number of lymphatic metastases and ANXA3 expression were identified as the independent risk factors affecting DFS and OS of patients with breast cancer, respectively. The relative risks of lymphatic metastasis and ANXA3 expression to DFS prognosis were 2.181 and 1.569, respectively, while those relative to OS prognosis were 2.052 and 1.698, respectively (Table 2).

## 4. Discussion

The present study evaluated the potential of ANXA3 as a biomarker for breast cancer. The findings positively correlated the expression of ANXA3 to lymphatic metastasis and histological grade independent of patient age, menopause status, tumor size, and clinical stage. Thereby, further studies on the prognostic value of ANXA3 in breast cancer are needed.

The major challenge in the therapies for cancer is the recognition of a stable biomarker with enhanced diagnostic efficacies. Identification of novel biomarkers will assist in developing efficient diagnostic procedures, thereby aiding prognosis and development of new molecules for targeted therapy. ANXA3 plays a significant role in tumorigenesis, resistance to chemotherapeutics, and subsequent metastasis [3]. An upregulation of ANXA3 by 63% was reported in patients with colorectal cancer [8] and a significant upregulation of ANXA3 was detected in the blood of colorectal cancer patients [9]. In patients with pancreatic cancer, the expression level of ANXA3 was 1.71 times higher than that in healthy individuals [10]. However, ANXA3 expression in prostate cancer tissue was significantly lower than that in benign prostate cancer tissue and high-grade prostatic intraepithelial neoplasia; an incomplete expression of ANXA3 protein was found in about 27.2% of patients, indicating that downregulated ANXA3 could promote occurrence and development of prostate cancer [11]. ANXA3 expression in lung cancer tissue with lymphatic metastasis was detected to be significantly higher in comparison to lung cancer tissue with the absence of lymphatic metastasis [12]. Also, ANXA3 expression was upregulated by 2.3 times in hepatocarcinoma cell lines Hca-F with high metastatic potential as compared to hepatocarcinoma cell lines Hca-P with low metastatic potential, indicating its involvement in hepatocarcinoma metastasis [13].

In the current study, differential expression of ANXA3 in different subtypes of breast cancer was detected by immunohistochemistry. The ANXA3 expression rate in breast cancer tissues was 64.25%. The highest expression of ANXA3 was detected in triple-negative breast cancer with poor prognosis as confirmed by the subsequent follow-up studies. Similar upregulation of the expression of ANXA3 is reported in prostate, ovarian, liver, rectal, and gastric cancers [10, 12, 24–27]. A positive correlation between the expression of annexin and breast cancer was established. Also, the clinicopathological analysis confirmed that the elevated levels of ANXA3 were significantly (*P* > 0.05) not associated with age, menopausal status, tumor size, and clinical stage; instead there was a significant (*P* < 0.05) correlation with lymphatic metastasis and histological grade. The data indicate a potential role of ANXA3 in promoting metastasis in breast cancer. Differential expression of ANXA3 can either promote or suppress the process of tumorigenesis depending on the cell type and the tissue involved [7]. These findings corroborate with earlier reports wherein a positive correlation of ANXA3 with axillary lymphatic metastasis was observed [28–30] and with Zeidan et al. who demonstrated that ANXA3 was a potential therapeutic target for breast cancer [31].

In the present study, we showed that the expression of ANXA3 was not correlated with the expressions of ER, PR, HER-2, and Ki-67. However, analysis of ANXA3 expression in breast cancers of different molecular subtypes revealed significantly (*P* = 0.026) higher levels of ANXA3 in triple-negative breast cancer compared to those in the three other groups (Luminal A, Luminal B, and HER-2 positive types). Triple-negative breast cancer has a poor prognosis compared to other major subtypes of breast cancer [32–34]. Also, poorer prognosis for the triple-negative breast cancer was observed in the follow-up procedures. Therefore, the findings are supportive for the correlation of ANXA3 expression with prognosis in breast cancer. Also, during the follow-up, it was observed that patients with a low level of ANXA3 expression exhibited superior DFS and OS compared to patients with a high level of ANXA3 expression. These observations indicate that ANXA3 may participate in occurrence, development, and metastasis of breast cancer.

The expression of ANXA3 is reported as an independent factor in the prognosis of prostate cancer and other cancers [11]. Hence, combined with our observation, ANXA3 can be viewed as a potential biomarker for the determination of infiltration, metastasis, and prognosis of breast cancer. However, the underlying mechanisms of ANXA3 in promoting proliferation and metastasis of tumor cells at the molecular level need further evaluation.

### 4.1. Limitations

Nevertheless, this study encounters limitations, especially with the difficulties involved in the follow-up. The follow-up duration was short, and patients of both the ANAXA3-positive and ANAXA3-negative groups were not followed up to a median survival time. In addition, due to limitations in the sample size, the pathological data can only be qualitatively classified into positive and negative results rather than being quantified. However, with recent developments in the diagnostic kits, ELISA can now be employed to determine the serum ANXA3 levels. Therefore, analysis of serum samples from the patients may further assist in verifying the findings of this report.

## 5. Conclusion

Increased expression of ANXA3 is associated with prognosis of breast cancer. The expression profile varies with the subtype of breast cancer; the highest expression levels were detected in patients with triple-negative breast cancer. The study, therefore, associates ANXA3 with prognosis and indicates its possible role in the occurrence, development, and metastasis of breast cancer.

## Figures and Tables

**Table 1:** Relationship between ANXA3 expression and clinical characteristics in breast cancer tissue.

	Number of cases (*n*)	ANXA3-negative (*n*)	ANXA3-positive (*n*)	ANXA3 positive rate (%)	*χ* ^2^	*P* value
Age (years)					0.365	0.546
<50	145	57	88	60.69		
≥50	164	59	105	64.02		
Menopausal status					1.560	0.240
Premenopausal	158	54	104	65.82		
Postmenopausal	151	62	89	58.94		
Tumor size (cm)					1.532	0.465
≤2	117	39	78	66.67		
>2, ≤5	160	65	95	59.37		
>5	32	12	20	62.50		
Clinical stage					0.607	0.738
I	54	20	34	62.96		
II	221	79	142	64.25		
III	34	10	24	70.59		
Molecular subtyping					9.226	0.026
Lu A	65	27	38	58.46		
Lu B	148	62	86	58.11		
HER-2 type	37	15	22	59.46		
Triple-negative	59	12	47	79.66		
Lymphatic metastasis					11.123	0.004
0	129	62	67	51.94		
1–3	108	35	73	67.59		
>3	72	19	53	73.61		
Histological grade					16.686	<0.001
I	35	24	11	31.42		
II	192	67	125	65.10		
III	82	25	57	69.51		

**Table 2:** Multivariate Cox analysis for factors affecting DFS and OS of breast cancer patients.

Factor	Disease-free survival		Overall survival	
	Hazard ratio (95% CI)	*P* value	Hazard ratio (95% CI)	*P* value
Age	1.135 (0.716, 1.798)	0.591	0.992 (0.650, 1.512)	0.969
Tumor size	0.818 (0.542,1.233)	0.337	0.877 (0.593, 1.298)	0.512
TNM stage	0.754 (0.367, 1.552)	0.444	0.898 (0.439, 1.838)	0.769
Oncology grade	1.441 (0.935, 2.221)	0.098	1.099 (0.753, 1.605)	0.625
Lymphatic metastasis	2.181 (1.425, 3.338)	<0.001	2.052 (1.370,3.073)	<0.001
Molecular subtyping	1.066 (0.812, 1.400)	0.645	1.074 (0.822, 1.404)	0.600
ANXA3 expression	1.569 (1.152, 2.138)	0.004	1.698 (1.240, 2.326)	0.001

## References

[1] Schlaepfer D. D., Haigler H. T. (1990). Expression of annexins as a function of cellular growth state. *Journal of Cell Biology*.

[2] Flower R. J., Perretti M. (2015). ‘Annexins’ themed section. *British Journal of Pharmacology*.

[3] Gerke V., Moss S. E. (2002). Annexins: from structure to function. *Physiological Reviews*.

[4] Swairjo M. A., Seaton B. A. (1994). Annexin structure and membrane interactions: a molecular perspective. *Annual Review of Biophysics and Biomolecular Structure*.

[5] Mussunoor S., Murray G. I. (2008). The role of annexins in tumour development and progression. *Journal of Pathology*.

[6] Protzel C., Richter M., Poetsch M. (2011). The role of annexins I, II and IV in tumor development, progression and metastasis of human penile squamous cell carcinomas. *World Journal of Urology*.

[7] Wu N., Liu S., Guo C., Hou Z., Sun M.-Z. (2013). The role of annexin A3 playing in cancers. *Clinical and Translational Oncology*.

[8] Madoz-Gúrpide J., López-Serra P., Martínez-Torrecuadrada J. L., Sánchez L., Lombardía L., Casal J. I. (2006). Proteomics-based validation of genomic data. *Molecular and Cellular Proteomics*.

[9] Yip K. T., Das P. K., Suria D., Lim C. R., Ng G. H., Liew C. C. (2010). A case-controlled validation study of a blood-based seven-gene biomarker panel for colorectal cancer in Malaysia. *Journal of Experimental and Clinical Cancer Research*.

[10] Baine M. J., Chakraborty S., Smith L. M. (2011). Transcriptional profiling of peripheral blood mononuclear cells in pancreatic cancer patients identifies novel genes with potential diagnostic utility. *PLoS ONE*.

[11] Köllermann J., Schlomm T., Bang H. (2008). Expression and Prognostic Relevance of Annexin A3 in Prostate Cancer. *European Urology*.

[12] Liu Y. F., Xiao Z. Q., Li M. X. (2009). Quantitative proteome analysis reveals annexin A3 as a novel biomarker in lung adenocarcinoma. *Journal of Pathology*.

[13] Liang R. C., Neo J. C., Ling Lo S., San Tan G., Keong Seow T., Chung M. C. (2002). Proteome database of hepatocellular carcinoma. *Journal of Chromatography. B, Analytical Technologies in the Biomedical and Life Sciences*.

[14] Siegel R., Naishadham D., Jemal A. (2012). Cancer statistics, 2012. *CA Cancer Journal for Clinicians*.

[15] Singletary S. E., Allred C., Ashley P. (2003). Staging system for breast cancer: revisions for the 6th edition of the AJCC Cancer Staging Manual. *Surgical Clinics of North America*.

[16] Wittliff J. L. (1984). Steroid hormone receptors in breast cancer. *Cancer*.

[17] Helin H. J., Helle M. J., Kallioniemi O.-P., Isola J. J. (1989). Immunohistochemical determination of estrogen and progesterone receptors in human breast carcinoma. Correlation with histopathology and DNA flow cytometry. *Cancer*.

[18] Sung C. O., Han S. Y., Kim S.-H. (2011). Low expression of claudin-4 is associated with poor prognosis in esophageal squamous cell carcinoma. *Annals of Surgical Oncology*.

[19] Shak S. (1999). Overview of the trastuzumab (Herceptin) anti-HER2 monoclonal antibody clinical program in HER2-overexpressing metastatic breast cancer. Herceptin multinational investigator study group. *Seminars in Oncology*.

[20] Singh K., Tantravahi U., Lomme M. M., Pasquariello T., Steinhoff M., Sung C. J. (2016). Updated 2013 College of American Pathologists/American Society of Clinical Oncology (CAP/ASCO) guideline recommendations for human epidermal growth factor receptor 2 (HER2) fluorescent in situ hybridization (FISH) testing increase HER2 positive and HER2 equivocal breast cancer cases; retrospective study of HER2 FISH results of 836 invasive breast cancers. *Breast Cancer Research and Treatment*.

[21] Wolff A. C., Hammond M. E. H., Hicks D. G. (2014). Recommendations for human epidermal growth factor receptor 2 testing in breast cancer: American society of clinical oncology/college of American pathologists clinical practice guideline update. *Archives of Pathology & Laboratory Medicine*.

[22] Wolff A. C., Hammond M. E. H., Schwartz J. N. (2007). American Society of Clinical Oncology/College of American Pathologists guideline recommendations for human epidermal growth factor receptor 2 testing in breast cancer. *Archives of Pathology & Laboratory Medicine*.

[23] Schluter C., Duchrow M., Wohlenberg C. (1993). The cell proliferation-associated antigen of antibody Ki-67: a very large, ubiquitous nuclear protein with numerous repeated elements, representing a new kind of cell cycle-maintaining proteins. *Journal of Cell Biology*.

[24] Bianchi C., Bombelli S., Raimondo F. (2010). Primary cell cultures from human renal cortex and renal-cell carcinoma evidence a differential expression of two spliced isoforms of annexin A3. *American Journal of Pathology*.

[25] Yan X., Yin J., Yao H., Mao N., Yang Y., Pan L. (2010). Increased expression of annexin A3 is a mechanism of platinum resistance in ovarian cancer. *Cancer Research*.

[26] Shekouh A. R., Thompson C. C., Prime W. (2003). Application of laser capture microdissection combined with two-dimensional electrophoresis for the discovery of differentially regulated proteins in pancreatic ductal adenocarcinoma. *Proteomics*.

[27] Tong S.-W., Yang Y.-X., Hu H.-D. (2012). Proteomic investigation of 5-fluorouracil resistance in a human hepatocellular carcinoma cell line. *Journal of Cellular Biochemistry*.

[28] Zeng C., Ke Z., Song Y. (2013). Annexin A3 is associated with a poor prognosis in breast cancer and participates in the modulation of apoptosis in vitro by affecting the Bcl-2/Bax balance. *Experimental and Molecular Pathology*.

[29] Yadwinder D. S., McDermott S., Lubman D. M. (2014). Annexin A3 is selectively expressed in MET-like as compared to EMT-like breast cancer stem cells. *Cancer Research*.

[30] Zeidan B. A., Jackson T., Larkin S. E. T. (2014). Annexin A3 is a breast cancer marker secreted by neoplastic cell lines and involved in cell migration, anticancer research. *Anticancer Research*.

[31] Zeidan B., Jackson T. R., Larkin S. E. T. (2015). Annexin A3 is a mammary marker and a potential neoplastic breast cell therapeutic target. *Oncotarget*.

[32] Boyle P. (2012). Triple-negative breast cancer: epidemiological considerations and recommendations. *Annals of Oncology*.

[33] Anders C., Carey L. A. (2008). Understanding and treating triple-negative breast cancer. *Oncology (Williston Park)*.

[34] Brouckaert O., Wildiers H., Floris G., Neven P. (2012). Update on triple-negative breast cancer: Prognosis and management strategies. *International Journal of Women's Health*.

